# Chronic stress promotes gastric cancer progression and metastasis: an essential role for ADRB2

**DOI:** 10.1038/s41419-019-2030-2

**Published:** 2019-10-17

**Authors:** Xuan Zhang, Yi Zhang, Zhongyuan He, Kai Yin, Bowen Li, Lu Zhang, Zekuan Xu

**Affiliations:** 1 Department of General Surgery, The Affiliated Hospital of Xuzhou Medical University, No. 99, Huaihai West Road, Xuzhou, Jiangsu Province 221002 China; 2 Department of General Surgery, The First Affiliated Hospital of Nanjing Medical University, No. 300, Guangzhou Road, Nanjing, Jiangsu Province 210029 China; 3 Department of General Surgery, Affiliated Hospital of Nanjing University of Chinese Medicine, No. 155 Hanzhong Road, Nanjing, Jiangsu Province 210029 China; 4 Department of General Surgery, Affiliated Hospital of Jiangsu University, No. 438, Jiefang Road, Zhenjiang, Jiangsu Province 212013 China

**Keywords:** Gastric cancer, Experimental models of disease

## Abstract

An increasing number of studies indicate that adrenergic signalling plays a fundamental role in chronic stress-induced tumour progression and metastasis. However, its function in gastric cancer (GC) and its potential mechanisms remain unknown. The expression levels of β-adrenergic receptor (ADRB) in GC cell lines were examined by using real-time polymerase chain reaction (RT-PCR) and western blotting. The effects of β2 adrenergic receptor (ADRB2) activation and blockade were investigated in vitro in GC cells by using proliferation, migration, invasion, cell cycle and apoptosis assays. Chronic restraint stress (CRS) increased the plasma levels of catecholamines and cortisol and also induced progression and metastasis of GC in vivo. Furthermore, immunohistochemical staining and a TUNEL assay were employed to observe the regulation of cell viability in vivo. The expression levels of ADRB2 in 100 human GC samples were measured by RT-PCR and immunohistochemistry. The stress hormones epinephrine and norepinephrine significantly accelerated GC cell proliferation, invasion and viability in culture, as well as tumour growth in vivo. These effects were reversed by the ADRB antagonists propranolol and ICI118,551 (an ADRB2-specific antagonist). Moreover, the selective ADRB1 antagonist atenolol had almost no effect on tumour cell proliferation and invasion in vitro and in vivo. ADRB2 antagonists suppressed proliferation, invasion and metastasis by inhibiting the ERK1/2-JNK-MAPK pathway and transcription factors, such as NF-κB, AP-1, CREB and STAT3. Analysis of xenograft models using GC cells revealed that ADRB2 antagonists significantly inhibited tumour growth and metastasis, and chronic stress antagonized these inhibitory effects. In addition, chronic stress increased the expression of VEGF, MMP-2, MMP-7 and MMP-9 in transplanted tumour tissue, and catecholamine hormones enhanced the expression of metastasis-related proteins. The expression of ADRB2 was upregulated in tumour tissues and positively correlated with tumour size, histological grade, lymph node metastasis and clinical stage in human GC samples. Stress hormone-induced activation of the ADRB2 signalling pathway plays a crucial role in GC progression and metastasis. These findings indicate that ADRB2 signalling regulates GC progression and suggest β2 blockade as a novel strategy to complement existing therapies for GC.

## Introduction

Stress is an inevitable social and psychological state in our daily lives that changes neurochemistry and endocrine and immune functions by activating the sympathetic nervous system (SNS) and releasing neurotransmitters such as catecholamines^1^. Extensive research has confirmed the role of social psychology and biological behaviour in tumourigenesis^2–4^. The impact of stress on tumourigenesis in humans has also become increasingly concerning^5–7^.

Most internal organs are innervated by sympathetic and parasympathetic nerves. These nerves play a pivotal role in tissue homoeostasis through direct innervation and release of neurotransmitters such as catecholamines and acetylcholine. Several experimental animal studies have also shown that psychosocial factors, especially chronic stress, can modulate the growth and progression of certain tumours by inducing the release of neurotransmitters^8–10^. SNS fibres are activated during chronic stress and release catecholamine neurotransmitters, which act on adrenergic receptors to regulate cellular biological behaviour^11–14^. Gastric cancer (GC) patients, especially patients who are diagnosed at an advanced stage, rarely achieve optimistic prognoses^15^. Therefore, a more detailed understanding of the cellular and molecular mechanisms of the growth and spread of GC is needed for the development of treatment strategies to exploit and ameliorate GC. Monoamine oxidase A (MAOA) is a catecholamine neurotransmitter-degrading enzyme. MAOA is also expressed in the digestive tract, liver, prostate, kidney and uterus^16,17^. Recent studies have reported altered MAOA in clinical GC samples, suggesting that β-adrenergic signalling may play an important role in GC progression and metastasis^18,19^. Basic research suggests that the β-receptor signalling pathway is involved in the regulation of tumourigenesis and the progression of biological processes^20,21^. However, the role of psychosocial factors, nerves and neurotransmitters in GC progression and metastasis has received little attention. Furthermore, the effect of stress-induced β-adrenergic signalling on cancer progression within the gastric microenvironment has not been investigated. In this study, we first demonstrated the stress-activated effects of the ADRB2 signalling pathway on the development and metastasis of GC from the following aspects: an in vitro cultured GC cell line, different chronic stress experimental models and clinical specimens.

## Results

### Expression of β-adrenergic receptor mRNA and protein in human GC and human normal epithelial cells

**Fig. 1:**
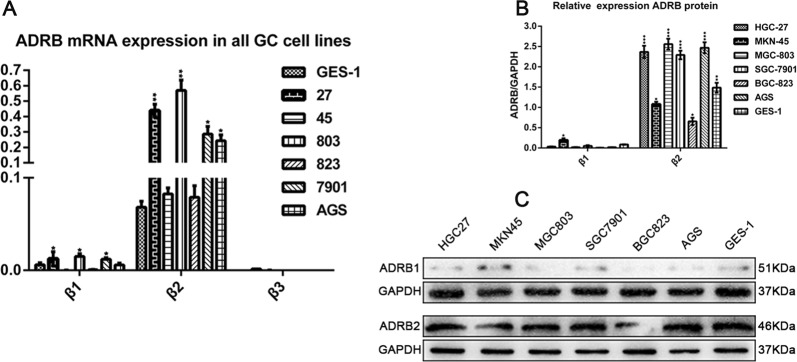
ADRB expression in human gastric tissues and GC cells. **a** qRT-PCR analysis of the ADRB mRNA levels in GC cell lines and GES-1 cells. **b**, **c** Western blotting was used to analyse the expression level of ADRB protein in GES-1 cells and GC cell lines. All data are presented as the mean ± SEM. *p < 0.05, **p < 0.01, or ***p < 0.001

Our results indicated that the human GC cell lines HGC27, MKN45, MGC803, BGC823, SGC7901, and AGS and a normal human gastric epithelial cell line (GES-1) express mRNA and protein for both ADRB1 and ADRB2 adrenergic receptors (Fig. 1). However, almost no expression of the ADRB3 adrenergic receptor was observed (Fig. 1a). RT-PCR (Fig. 1a) and western blotting (Fig. 1b, c) results jointly confirmed that the mRNA and protein expression of ADRB2 was approximately five-fold higher than that of ADRB1 in the four cell lines, particularly in the HGC27 and MGC803 cell lines. These results suggest that ADRB2 may be the most important adrenergic receptor in GC cell lines.

### Effect of β-adrenergic signalling on the proliferation of GC cells in vitro

The proliferation ability of GC cells was stimulated by epinephrine and norepinephrine to peak levels at the 10-μM concentration, whereas the effect was not significant at 50, 100 and 150 μM (Supplementary Fig. 1A–D). The data indicated that isoproterenol (a non-selective ADRB agonist), terbutaline (a selective ADRB2 agonist) and dobutamine (a selective ADRB1 agonist) were the most effective at stimulating the proliferation of GC cells at 1, 10 and 50 μM, respectively (Supplementary Fig. 1E, F, G). Exogenous ADRB blockers propranolol (a non-selective ADRB antagonist), atenolol (a selective ADRB1 antagonist), and ICI118,551 (a selective ADRB2 antagonist) inhibited proliferation of GC cells at a concentration of 50 μM but did not exert a stronger effect with an increase in drug concentrations (Supplementary Fig. 1H, I, J). The use of an ADRB2 agonist (terbutaline) to pretreat GC cells obviously attenuated the inhibitory effect of propranolol and ICI118,551, and this phenomenon was not pronounced in the ADRB1 agonist (dobutamine) group (Supplementary Fig. 3A, B).

**Fig. 2:**
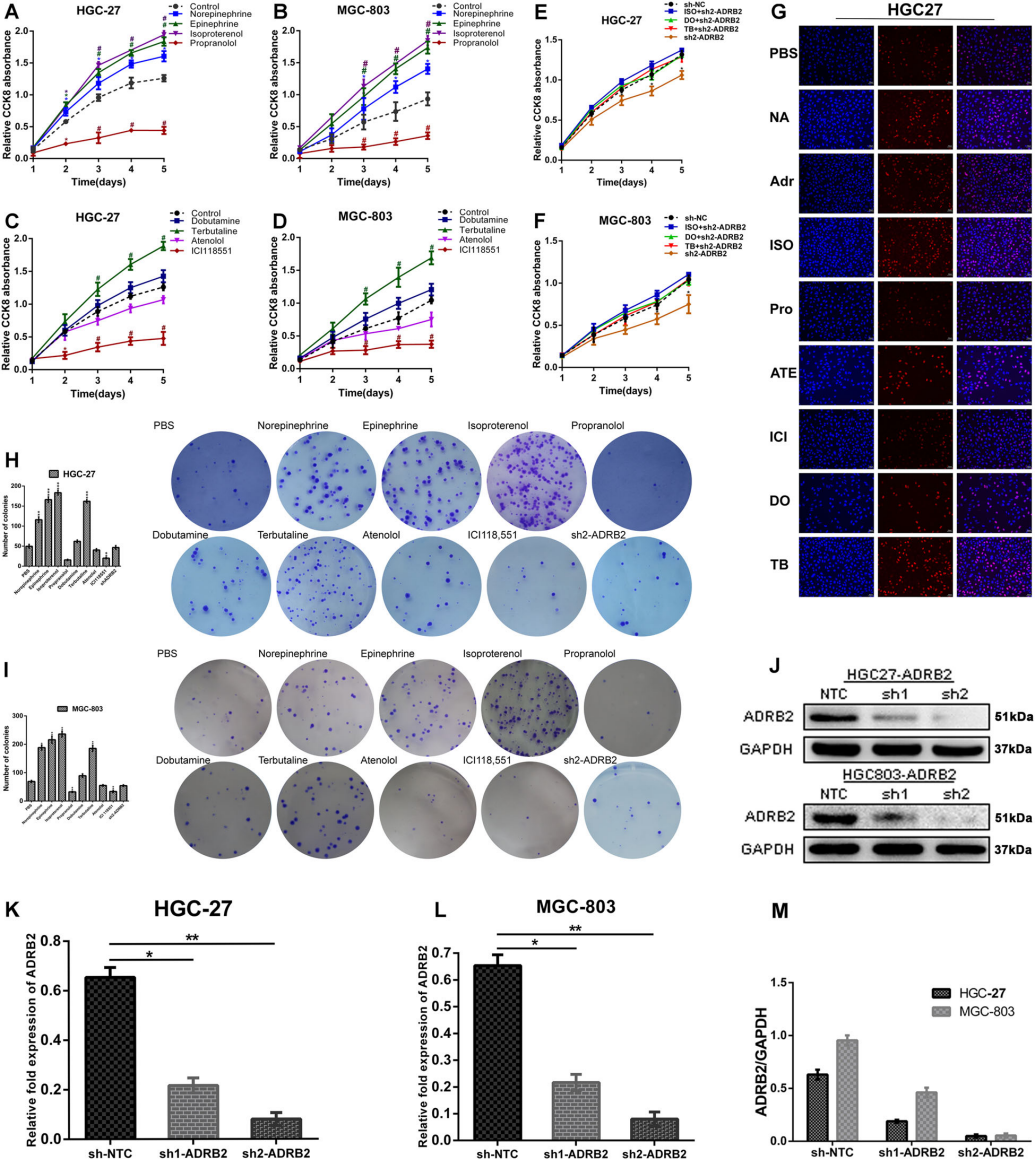
Effect of β-adrenergic signalling on the proliferation of GC cells in vitro. **a**, **b** Effects of non-specific ADRB agonists and blockers on HGC27 and MGC803 cell proliferative ability by CCK8 assay. **c**, **d** The effects of specific ADRB agonists and blockers on the proliferation potential of HGC27 and MGC803 cells were determined by a CCK8 assay and are presented as a growth curve. **e**, **f** Knockdown of ADRB2 expression, and CCK8 validation of various types of ADRB agonists on the proliferation of gastric cancer. **g** The EdU assay validated the functional role of various types of ADRB agonists and antagonists in HGC27 cell proliferation. **h**, **i** A colony-forming assay was used to detect the effects of various specific ADRB agonists and blockers on the colony-forming ability of HGC27 and MGC803 cells. Representative images are shown. **j**, **k**, **l**, **m** The efficiency of ADRB2 knockout was determined by real-time quantitative PCR and immunoblotting

We treated HGC27 and MGC803 cells with epinephrine (10 μM), norepinephrine (10 μM), isoproterenol (1 μM), dobutamine (50 μM) and terbutaline (10 μM) for 1, 2, 3, 4, or 5 days. Epinephrine, norepinephrine, isoproterenol and terbutaline markedly increased the viability of HGC27 and MGC803 cells (Fig. 2a–d). Consistently, a plate clone assay revealed that the colony formation ability of the two GC cell lines was obviously enhanced with epinephrine, norepinephrine, isoproterenol and terbutaline treatment (Fig. 2h, i). In contrast, treatment with dobutamine did not markedly affect cell proliferation (Fig. 2c, d, g). Furthermore, in an EdU incorporation assay, epinephrine, norepinephrine and terbutaline significantly promoted the proliferation of GC cells (Fig. 2g, Supplementary Fig. 2B). We next observed the effect of propranolol (50 μM) and ICI118,551 (50 μM) on the proliferation of GC cells. The results indicated that propranolol and ICI118,551 significantly decreased cell viability (Fig. 2a–d) and notably suppressed clonogenic survival (Fig. 2h, i). We also found that pretreatment of GC cell lines with propranolol and ICI118,551 attenuated the potentiation of catecholamine, and the inhibitory effect of propranolol was stronger than that of ICI118,551 (Supplementary Fig. 3H, I, J, K, L).

To further verify the function of ADRB2 in GC, we introduced the shADRB2 interference sequence or control shNTC into lentiviruses and infected cells to establish stable ADRB2 knockdown (HGC27 shADRB2 and MGC803 shADRB2) cell lines. The effect of ADRB2 knockdown in the cell lines by RT-PCR and western blotting is shown in Fig. 2j–m. The interference sequence reduced the mRNA level of ADRB2 to 10%. After confirming the knockdown effect, the two cell lines were treated with different types of ADRB agonists. The proliferative activity of each agonist group was significantly affected, and there was no significant difference between these groups and the control group (Fig. 2e, f). In the shADRB2 group, the ADRB2 agonist-mediated promotion of the proliferation effect was significantly different compared with that of the control group (Supplementary Fig. 3C, D).

### ADRB2 affects the activation of the transcription factors NF-κB, AP-1, CREB, and STAT3 and the ERK/JNK/ MAPK signalling pathway

Western blotting results showed that norepinephrine, epinephrine, isoproterenol and terbutaline significantly upregulated NF-κB expression levels and notably increased AP-1, CREB and STAT3 phosphorylation in HGC27 or MGC803 GC cells after cultivation with ADRB agonists for 48 h (Supplementary Fig. 3A–D). Conversely, phosphorylation of AP-1, CREB and STAT3 was decreased in HGC27 and MGC803 cells treated with ADRB blockers.

ADRB blocked the downregulation of p-ERK1/2 and p-JNK expression levels but did not change their total protein expression levels. In contrast, phosphorylation of ERK1/2 and JNK was obviously upregulated in HGC27 and MGC803 GC cells treated with ADRB agonists. Moreover, we used the ERK1/2- and JNK-specific inhibitors U0126 and SP600125, respectively, to treat HGC27 and MGC803 cell lines. The adrenaline + atenolol, adrenaline + ICI118,551, adrenaline + U0126 and adrenaline + SP600125 groups showed a dramatic reduction in the proliferation index of GC cells, compared with that of the ADRB agonist (adrenaline) group, suggesting that the ERK1/2-JNK-MAPK pathway plays a key role in promoting the growth of GC cells (Supplementary Fig. 2D, E, H). Furthermore, we used terbutaline to directly stimulate ADRB2 and ascertained that U0126 and SP600125 markedly lessened the effect of terbutaline (Supplementary Fig. 2F, G).

### β-adrenergic signalling regulates the migration and invasion potential of GC cells in vitro

**Fig. 3:**
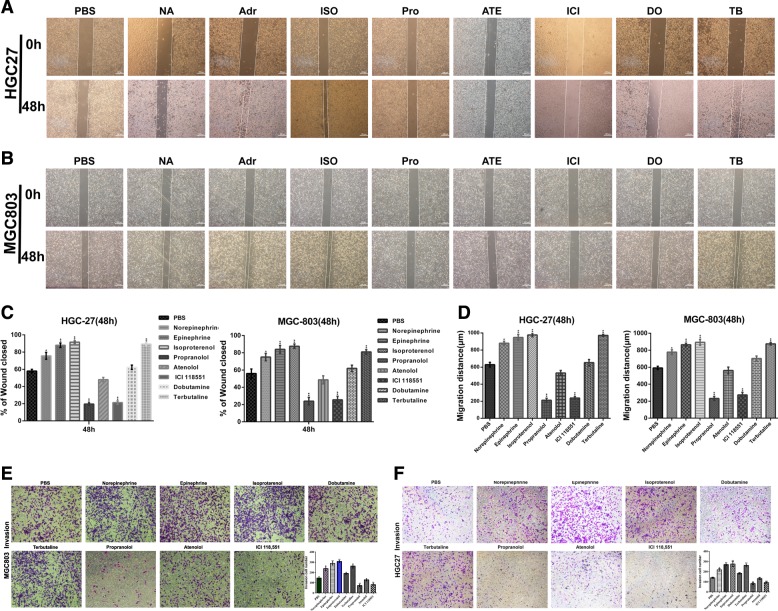
β-adrenergic signalling regulates the migration and invasion potential of GC cells in vitro. **a**–**d** Stress hormones and specific ADRB2 agonists promoted the migration of GC cells in a wound-healing assay. Propranolol and a specific ADRB2 antagonist attenuated wound healing in HGC27 and MGC803 cells. Original magnification, ×40; Scale bar = 100 μm. Photographs were taken immediately (0 h) and 48 h after wounding, and quantification of wound closure was performed. **e**, **f** An invasion assay was performed on each cell group. Original magnification, ×100; Scale bar = 100 μm

The wound scratch healing results indicated that stress hormones and specific ADRB2 agonists enhanced the flattening and spreading of GC cells (Fig. 3a–d). Transwell assays also demonstrated that catecholamines and terbutaline conspicuously increased the number of migrating and invading HGC27 and MGC803 cells (Fig. 3e, f).

Our data showed that the ADRB antagonist weakened the migration potential of HGC27 and MGC803 cells (Fig. 3a–d). Propranolol and ICI118,551 showed a significant decrease in the migration rate and markedly reduced the number of invading HGC27 and MGC803 cells (Fig. 3e, f).

### Blocking β-adrenergic signalling induces G1/S phase cell cycle arrest and apoptosis in GC cells

**Fig. 4:**
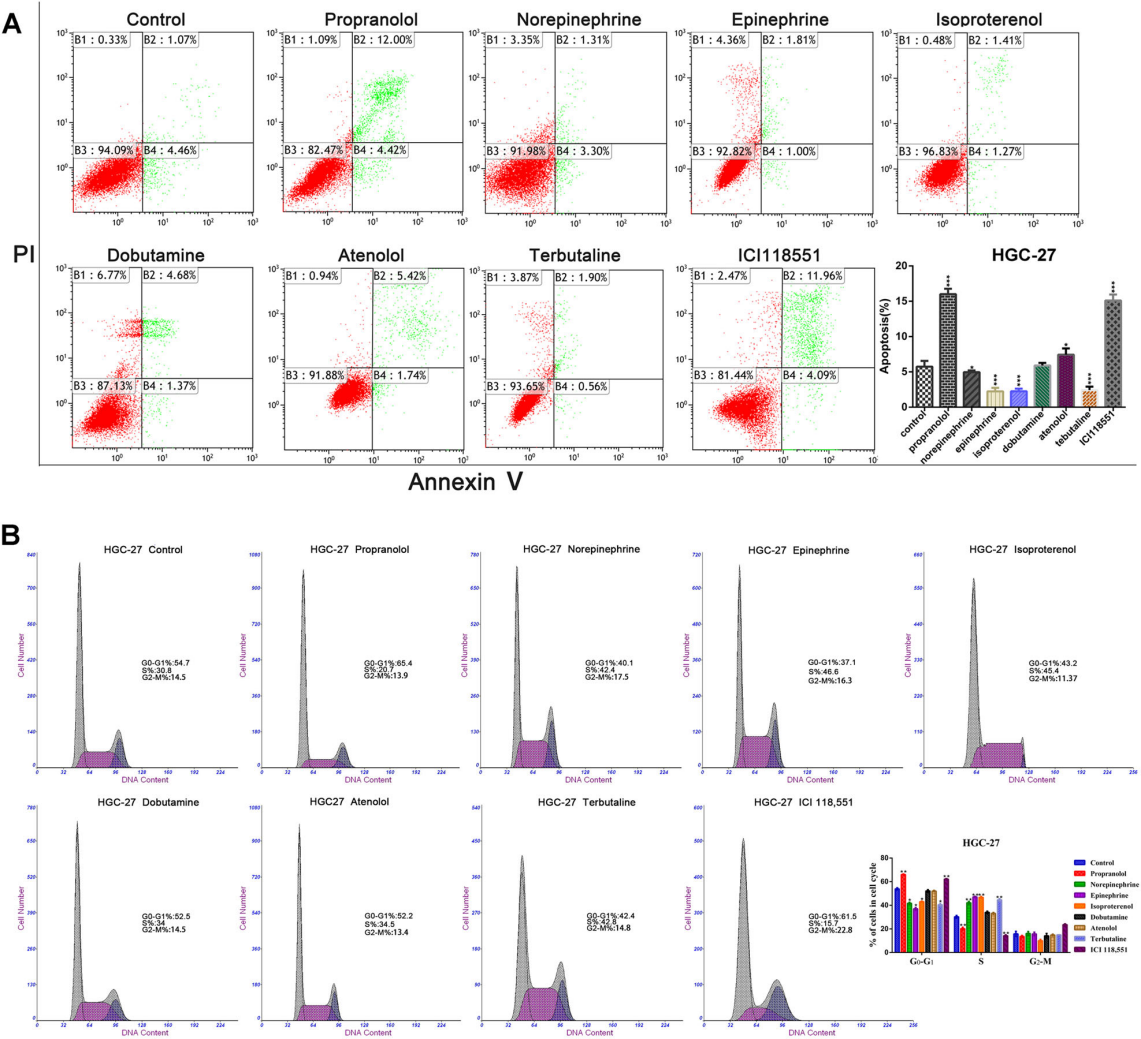
The effects of different subtypes of ADRB agonists and antagonists on cell cycle distribution and apoptosis of HGC27 GC cells. **a** The ADRB2-blocking group showed a specific loss of the ADRB2 signal in HGC27 cells, and the percentage of apoptotic cells was obviously increased, compared with those of the control and ADRB1-blocking groups. Furthermore, selective activation of ADRB2 significantly reduced the rate of apoptosis. **b** The percentages of G0-G1 phase cells in the population of cells treated with the specific ADRB2 antagonist ICI118,551 and non-specific ADRB antagonist propranolol were increased compared with those in cells treated with atenolol and the control. Moreover, the percentages of S phase cells were significantly reduced (*p* < 0.05)

Compared with percentages in the PBS- and atenolol-treated cells, propranolol and ICI118,551 significantly reduced the percentage of GC cells in S phase (Fig. 4b, Supplementary Fig. 5B). Western blot analysis illustrated that CyclinD1/CDK4/CDK6/p-Rb protein levels were notably increased in the epinephrine, isoproterenol and terbutaline groups (Supplementary Fig. 5D). Next, flow cytometry revealed that the percentage of apoptotic cells was evidently increased when ADRB2 expression was blocked in HGC27 and MGC803 cells (Fig. 4a, Supplementary Fig. 5A). Expression of the anti-apoptotic proteins Bcl-2 and Bcl-XL was upregulated by stress hormones and terbutaline, while the levels of cleaved caspase-3 and the pro-apoptotic protein Bax in GC cells were induced by non-selective ADRB blockers and specific ADRB2 blockers (Supplementary Fig. 5C). The ADRB1 agonist and ADRB1 blockers had no significant anti-apoptotic or pro-apoptotic effects.

### Effect of a simple chronic stress state on GC growth and upregulation of catecholamine levels in vivo

**Fig. 5:**
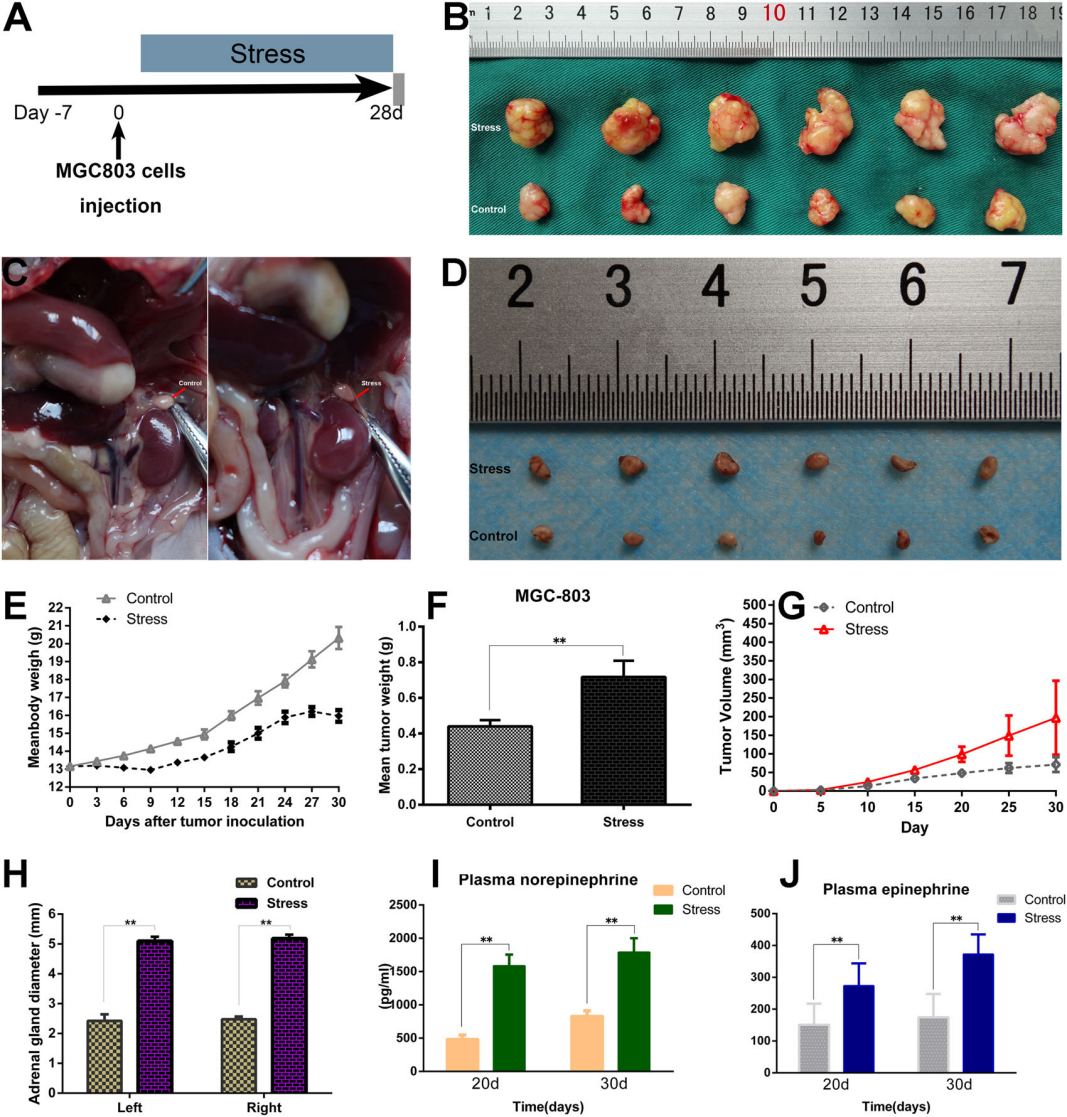
Chronic restraint stress protocol. **a** Seven days before the MGC803 cells were injected into the flanks of nude mice, the mice were exposed to chronic stress conditions (daily restraint) vs home cage control conditions for 8 h daily, lasting for 28 days. **b** Representative tumour images from the chronic stress group and the stress-free control group are displayed. **c**, **d** After 4 weeks, the adrenal gland diameters of the control group and the stress group were measured. **e** The weight curve of the two groups of nude mice is shown. **f** The mean tumour weight was monitored. **g** The volume of the tumour during the process of tumour growth at six different time points was determined. **h** Adrenal gland diameter quantitative results are shown. **i**, **j** The average levels of NA and Adr were analysed by ELISA

We first validated whether a simple chronic stress state affects tumour growth in vivo (Fig. 5a). During the observation of behavioural changes in mice, nude mice consumed less food, drank less water, showed reduced activity and lost weight in the persistent chronic stress state (Fig. 5e). The tumour weight of the chronic restraint stress group was remarkably higher than that of the control group (Fig. 5f). By contrast, the tumour volume of the control group was smaller than that of the chronic restraint stress group (Fig. 5b, g). Moreover, we used the adrenergic neurotoxic agent 6-OHDA to selectively destroy peripheral sympathetic nerves in mice. We found that the weight of each mouse and average weight of subcutaneous tumours in nude mice subjected to chronic restraint stress + 6-OHDA were not significantly different than those of the control groups (supplementary Fig. 6A-C).

In nude mice, the adrenal glands of the chronic stress group were evidently larger than the adrenal glands of the control group, suggesting that the HPA axis remained active in the chronic stress state (Fig. 5c, d, h). As illustrated in Fig. 5i, j, the concentrations of norepinephrine and epinephrine in the chronic stress group were demonstrably higher than those in the control group.

**Fig. 6:**
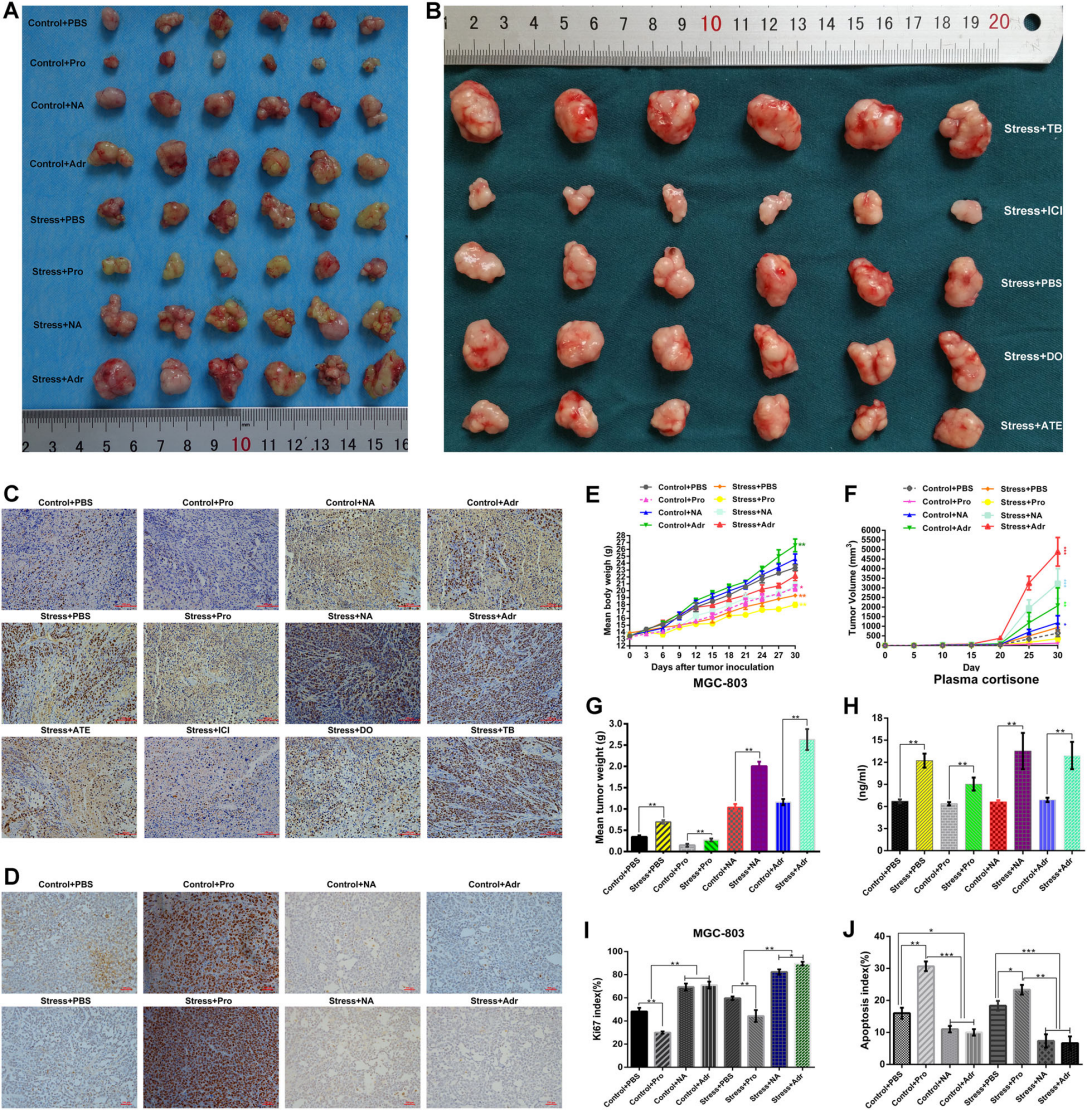
Chronic stress promotes gastric cancer progression. **a** MGC803-inoculated mice were treated with PBS, epinephrine, norepinephrine, or propranolol added to drinking water. Another four groups of mice were treated with chronic stress combined with drug treatment. **b** HGC27 cells were inoculated into the right flanks of nude mice 7 days after initiating stress. **c** Immunohistochemical staining targeting Ki-67. Original magnification, ×100; Scale bar = 100 μm. **d** TUNEL assay was used to detect apoptotic cells in tissues. Scale bar = 100 μm. **e** The body weight of each mouse in each experimental group was recorded during the trial. **f** Tumour volumes on the indicated days are presented as growth curves. **g** The mean tumour weight of each group was determined. **h** The data represent the average serum cortisone levels in each group. **i** The percentage of cells positive for Ki-67 staining is presented as a Ki-67 index. **j** Representative TUNEL-positive cells were used to draw the histogram

We also used immunohistochemistry to quantify the number of Ki-67-positive cells. Compared with levels in the control, chronic restraint stress notably promoted the growth of MGC803 cells in vivo as demonstrated by Ki-67 (Fig. 6c) and PCNA (Supplementary Fig. 2A, C) staining.

### Activation of β2-adrenergic signals can promote the growth of gastric cancer xenografts under chronic stress, whereas blockade of β2-adrenergic signals can inhibit this effect

The body weight growth curve of the stress group showed that the nutritional status of the body under chronic stress was affected (Fig. 6e). As shown in Fig. 6f, g, the average tumour volume curve and tumour weight in the stress group were higher than those in the control group. Propranolol interfered with the tumourigenic ability of GC cell lines and, to a certain extent, worked against the effect of chronic stress on the growth of transplanted tumours. Next, we observed the effects of various selective ADRB1 and ADRB2 agonists and blocking agents on tumourigenicity in nude mice. Terbutaline showed obvious promotion of tumourigenicity, and ICI118,551 noticeably inhibited oncogenicity in mice. The selective ADRB1 agonist showed a poor ability to promote tumour capacity that was not considerably different from that of the control group (Fig. 6b). The Ki-67 proliferation index of each stress group was higher than that of the control group (Fig. 6c, i).

### In vivo analysis of the role of β-adrenergic signalling in regulating cell viability and metastasis

TUNEL staining of tumour specimens revealed that propranolol increased the percentage of apoptotic cells in the transplanted tumour, inhibited the proliferation of GC cells in vivo, and induced apoptosis of GC cells (Fig. 6d, j). In contrast, stress hormones drastically inhibited apoptosis in transplanted tumours and promoted the proliferation of GC cells (Fig. 6d, j).

**Fig. 7:**
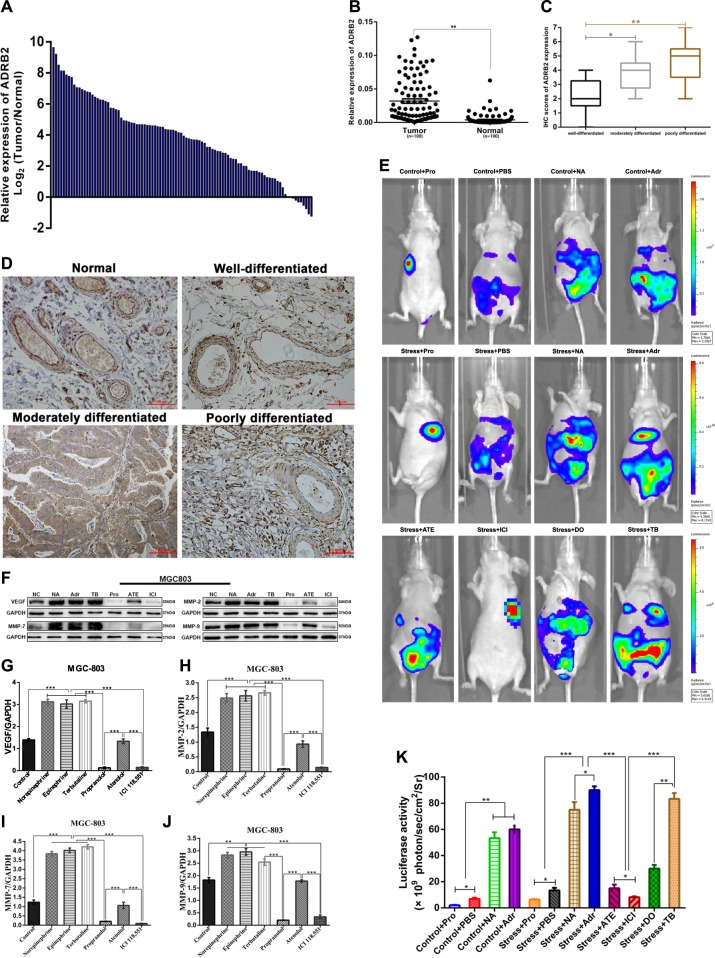
In vivo analysis of ADRB2-mediated regulation of GC growth and metastasis. **a** The relative expression of ADRB2 in gastric cancer tissues compared with that of the corresponding adjacent normal tissues. ADRB2 expression was examined by qPCR and normalized to GAPDH expression. **b** The expression of ADRB2 was detected in 100 paired GC tissues and adjacent tissues. **c** Box plot showing the IHC scores for ADRB2 protein expression in human GC tissues. **d** Immunohistochemical staining of ADRB2 in human normal gastric tissue and tumour tissues. **e** Photographs of tumours were taken by the IVIS Imaging System. **f**, **g**, **h**, **i**, **j** The expression of VEGF, MMP-2, MMP-7 and MMP-9 in tumour samples. All data are presented as the mean ± SEM. *p < 0.05, **p < 0.01, or ***p < 0.001

Chronic stress increased the metastatic ability of primary GC cells to migrate to distant tissues; adrenaline, norepinephrine, and terbutaline enhanced this effect (Fig. 7e, k). Moreover, propranolol inhibited the metastasis of GC cells in nude mice; in vivo optical imaging showed chemiluminescence intensity values were markedly lower than those in the epinephrine and norepinephrine groups, and a similar phenomenon was observed in the ICI118,551 group (Fig. 7e, k).

As shown in Fig. 7f–j, ADRB agonists can promote the expression of VEGF, MMP-2, MMP-7 and MMP-9 protein in vivo. In addition, the expression levels of VEGF, MMP-2, MMP-7 and MMP-9 were lower after treatment with ADRB blockers than after treatment with the control, and ICI118,551 and propranolol showed a greater inhibitory effect than did atenolol.

### The expression level of ADRB2 in GC tissues is correlated with tumour size, histological grade, lymph node metastasis and clinical stage

We divided the specimens collected from 100 (53 men and 47 women) GC patients into groups, including GC tissues and adjacent normal tissues. GC tissues with higher than the median expression of ADRB2 were assigned to the high group, while those with less than the median expression of ADRB2 were assigned to the low group. ADRB2 mRNA expression was drastically higher in GC tissues than that in matched non-cancerous tissues (Table 1, Fig. 7a). As shown in Table 1, patients with high levels of ADRB2 had significantly increased tumours, reduced histological grades and later clinical stages (Table 1). Our data also indicated that ADRB2 levels were upregulated in GC tissues and highly correlated with lymph node metastasis (Table 1). However, there was no notable difference in ADRB2 expression levels between age, sex, and T classification. Next, we found that ADRB2 protein expression was low in normal gastric tissue but aberrantly elevated in GC tissues, especially in poorly differentiated tissues (Fig. 7b, c, d).

**Table 1:** Correlation of relative ADRB2 expression with the clinicopathological characteristics of patients with GC

Characteristics		Total no. of patients	ADRB2 expression		*P* value
			High group	Low group	
Age (years)	≥60	65 (65%)	50	15	0.545
	<60	35 (35%)	25	10	
Gender	Male	53 (53%)	39	14	0.728
	Female	47 (47%)	36	11	
Tumour size (cm)	≥3.5	60 (60%)	50	10	0.018*
	<3.5	40 (40%)	25	15	
Histological grade	Well-moderately	48 (48%)	29	19	0.001*
	Poorly-signet	52 (52%)	46	6	
Clinical stage	I–II	39 (40%)	35	4	0.006*
	III–IV	61 (60%)	40	21	
T classification	T1–T2	31 (31%)	19	12	0.033*
	T3–T4	69 (69%)	56	13	
Lymph node metastasis	Negative	42 (42%)	24	18	0.000*
	Positive (N1–N3)	58 (58%)	51	7	
Liver metastasis	Absent	91 (91%)	70	21	0.157
	Present	9 (9%)	5	4	

Correlation was estimated by the Fisher’s exact test, *p <0.05 statistically significant difference

## Discussion

**Fig. 8:**
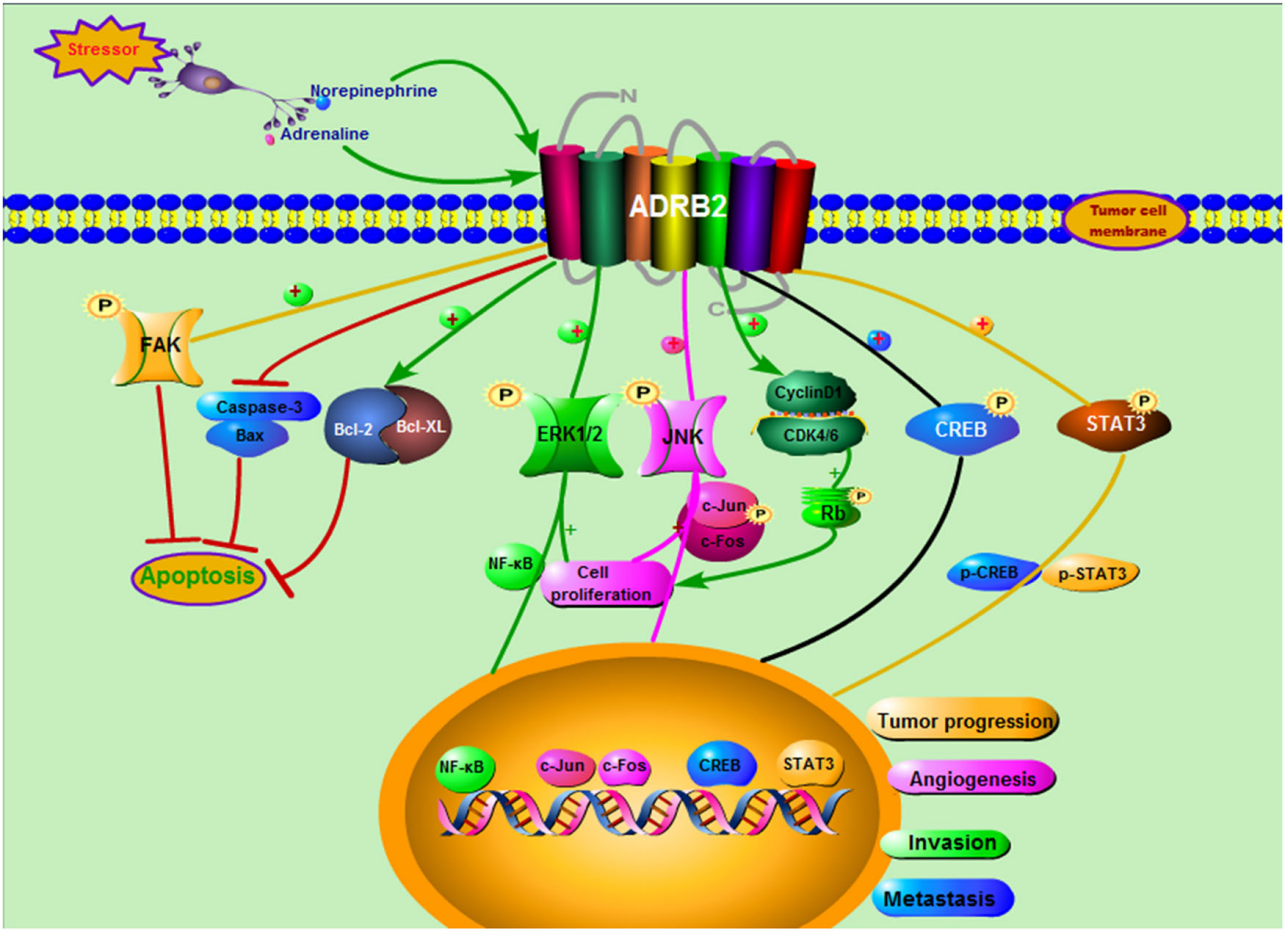
Schematic representation of the proposed mechanism of ADRB2-modulated GC progression and metastasis

In this study, we concluded that (Fig. 8) sympathetic innervation is positively correlated with the progression of GC and that sympathetic neurotransmitters promoted the malignant biological behaviour of GC cells by activating ADRB2. Our results reveal the supportive role of sympathetic innervation in the pathogenesis of GC and suggest ADRB2 as a potential therapeutic target for the treatment of GC. Furthermore, in addition to the sympathetic nervous system, the parasympathetic nervous system (as the other division of the autonomic nervous system) has also been proposed to play a key role in tumour progression^22,23^. Sympathetic and parasympathetic nerves have been shown to infiltrate the tumour microenvironment and actively stimulate cancer cell growth and dissemination^24^. This mechanism involves the release of neurotransmitters, such as catecholamines and acetylcholine, directly into the vicinity of cancer and stromal cells to activate corresponding membrane receptors.

In recent years, the role of nervous system regulation in the occurrence and development of cancer has gradually been widely studied and recognized^25–27^. Traditionally, the idea that there is no nerve distribution in cancer tissue has been changed with expanding research. Many studies have confirmed that neuronal innervation of tumours, as one of the tumour microenvironment components, plays an important role in the occurrence and development of tumours^12,28,29^. Seifert et al.^30^ used immunohistochemical staining to detect a neuron-like structure with vasoactive intestinal peptide activity in bladder cancer tissue. Feng et al.^31^ found that there were nerve fibres in GC tissues, and the density of the nerve fibres was linked to the degree of differentiation of GC. After sympathetic and parasympathetic excitation, neurotransmitters, such as adrenaline, acetylcholine, and substance P, are released through their nerve endings. These neurotransmitters activate receptors and then cause downstream signal transduction by binding to specific receptors on the cell surface. The stomach is likewise dominated by sympathetic and parasympathetic nerves. It has been reported that denervation can inhibit the occurrence of GC, which is linked to inhibition of the Wnt signalling pathway and the expansion of cancer stem cells^32^. In addition, activation of muscarinic receptors has been proven to promote cell transformation and cancer progression. Previous studies from our laboratory have shown that M3 receptor expression is elevated in GC and is associated with cancer stage and lymph node metastasis^33^. This study is a powerful complement to the theory of autonomic nervous system regulation of gastric cancer progression.

We further confirmed that the ADRB agonist binds to the β receptor on the cell membrane and activates adenylate cyclase to increase intracellular cAMP concentration. As the second messenger of the ADRB signalling pathway, cAMP can further activate and phosphorylate protein kinase A (PKA). Activated PKA can activate a series of transcription factors (including CREB and NF-κB), leading to cell proliferation. Almost all tumours are deficient in cell cycle regulation, and cell cycle failure can induce cancer by promoting cell proliferation^34,35^. Similar to other studies^36^, our experimental results also indicated that ADRB blockers can inhibit tumour cell growth and induce G1/S arrest. CyclinD1 is an important regulator of G1 phase progression to S phase in the cell cycle. In addition, CyclinD1 binding with CDK4/6 activated CDK4/6 and catalysed Rb phosphorylation. Our experimental data show that ADRB antagonists can significantly downregulate CyclinD1 expression and inhibit the formation of the CyclinD1/CDK4/6 complex, leading to G1/S arrest. In addition to cell cycle regulation, apoptosis is an important way to regulate the survival of tumour cells. Moreover, the proportion of viable cells among GC cells decreased, and the percentage of apoptotic cells increased after blockade of ADRB2 signalling and knockdown of ADRB2 expression.

In the mouse stress model, we found that restraint stress increased the levels of catecholamine hormones and glucocorticoids in the circulation. To assess the role of the sympathetic nervous system in GC, we used the adrenergic neurotoxin 6-hydroxydopamine to selectively damage the peripheral sympathetic nerves of mice. The growth of subcutaneously transplanted tumours was slowed under stress, and there was no significant difference in volume and average weight between the two groups. There were no significant differences in serum catecholamine hormones and glucocorticoid levels between the two groups of mice. These results indicate that peripheral sympathetic nerve lesions can reverse the adverse effects of chronic stress on the body and attenuate stress-mediated tumourigenic effects in vivo. It is confirmed that sympathetic nerve activity plays a major role in promoting the growth of GC.

The polarization of macrophages (TMEs) in the tumour microenvironment to M2 is another important pathway for tumour progression^3,37,38^. It has been observed that adrenaline can promote the transformation of macrophages from M1 to M2 and promote the progression of cancer by influencing macrophages in the tumour microenvironment^39^. This effect is mediated by ADRB2. Blocking ADRB2 activity in breast cancer patients may inhibit tumour progression by reversing M2 macrophage polarization. Tumours induce angiogenesis under the action of pro-angiogenic factors in their microenvironment to facilitate their distant metastasis. Sympathetic neurotransmitters released into the blood under chronic stress can induce related signal transduction and stimulate the synthesis and secretion of VEGF and other related pro-angiogenic growth factors in different types of malignant tumours^40^. In our experiments, we confirmed that the protein expression of VEGF, MMP-2, MMP-7 and MMP-9 in mouse GC tissue was significantly increased after activation of ADRB signalling under the condition of chronic stress; thus, these proteins play a role in promoting cancer and metastasis. The in vivo metastasis model further confirmed that activation of the ADRB2 signalling pathway in chronic stress could significantly promote liver and lung metastasis of GC cells in nude mice. We also found that propranolol can inhibit the metastasis of gastric cancer cells in vivo. In conclusion, these results suggest that chronic stress mainly promotes metastasis and invasion of gastric cancer cells through ADRB2 in vivo.

Some studies have described the upregulation of ADRB2 expression in different tumours with clinical and pathological findings^41–43^. These studies indicate that the prognostic significance of ADRB2 expression appears to be due to different histological types of tumours. In patients with oral squamous carcinoma, the expression of ADRB2 was weak/negative, and the prognosis was poor. However, the increased expression of ADRB2 in patients with non-squamous cell carcinoma is significantly associated with a shorter survival period. In this study, we examined the expression levels of ADRB2 mRNA in 100 pairs of GC tissues and matched tumour-adjacent tissues. The expression level of ADRB2 in GC specimens was significantly higher than that in normal gastric tissues. Positive expression of ADRB2 was related to tumour size, poor differentiation, late clinical stage and lymph node metastasis. These results support the hypothesis that ADRB2 may play a key role in the carcinogenesis of GC, and the overexpression of ADRB2 may play an important role in the development and prognosis of GC tumours. Therefore, ADRB2 may be a potential prognostic biomarker of GC.

Our study has some limitations. At present, all studies are based on the transplanted tumour model in nude mice, which can only explain the role of stress in the progression of GC. However, the role of chronic stress in the occurrence of GC remains unknown. In addition, the current nude mouse transplanted tumour model does not include the tumour immune microenvironment, and the role of chronic stress-induced immunosuppression in promoting the tumour immune escape mechanism has not yet been elucidated. Therefore, the current results have some limitations.

Taken together, our data indicate that the ADRB2 signalling pathway can enhance tumourigenesis, angiogenesis and metastasis in GC cell lines under chronic stress. These data provide evidence and a basis for a better understanding of the effects of external environmental factors on GC progression and suggest that ADRB2 blockers can be used to clinically control GC progression and improve patient outcomes.

## Materials and methods

### Cell culture and RT-PCR

Human normal gastric epithelial cell lines (GES-1) and human GC cell lines (BGC823, HGC27, MKN45, SGC7901, AGS and MGC803) were obtained from the Cell Bank of the Chinese Academy of Medical Science (Shanghai, China). These cells were cultured in Roswell Park Memorial Institute (RPMI)-1640 medium containing 10% foetal bovine serum (Invitrogen Life Technology, CA, USA), penicillin (100 U/ml), and streptomycin (100 mg/ml) at 37 °C with 5% CO_2_. Different concentrations of epinephrine, norepinephrine, isoproterenol, terbutaline, dobutamine, propranolol, atenolol and ICI118,551 (0, 0.1, 1, 10, 50, 100 and 150 μM) were then incubated with highly expressing ADRB2 GC cell lines for 72 h to investigate the effects of exogenous ADRB agonists and antagonists at different concentrations on the proliferation of GC cell lines by the CCK8 method. Epinephrine, norepinephrine, isoproterenol, terbutaline, dobutamine, propranolol, atenolol and ICI118,551 were purchased from Sigma-Aldrich (St. Louis, MO, USA).

Total cellular RNA was isolated using TRIzol (Invitrogen Life Technology, CA, USA), and cDNA was synthesized using PrimeScript RT reagent (Takara, Dalian, China) according to the manufacturer’s protocol. cDNA was amplified by Power SYBR Green PCR master mix (Applied Biosystems, Foster City, USA) according to the manufacturer’s protocol in an Applied Biosystems 7500 sequence detection system. The levels of gene expression were determined by the ΔΔCT method, and the results were expressed as mRNA expression levels normalized to the levels of β-actin. The qRT-PCR primers used were as follows: ADRB1, 5′-CTCCTTCTTCTGCGAGCTGTGGA-3′ (sense) and 5′-ATGAGGATGGGCAGGAAGGACA-3′ (antisense); ADRB2, 5′-TTGCTGGCACCCAATAGAAGC-3′ (sense) and 5′-CAGACGCTCGAACTTGGCA-3′ (antisense); ADRB3, 5′-GCCCAATACCGCCAACAC-3′ (sense) and 5′-GCCAGCGAAGTCACGAACAC-3′ (antisense); and β-actin, 5′-TTAGTTGCGTTACACCTTTC-3′ (sense) and 5′-ACCTTCACCGTTCCAGTTT-3′ (antisense). Each PCR analysis was performed in triplicate and independently repeated three times.

### Cell proliferation and colony formation assay

Cell proliferation was measured using CCK8 (Dojindo, Japan) and 5-ethynyl-20-deoxyuridine (EdU, C10301, RiboBio) according to the manufacturer’s specifications. Cells treated with various ADRB agonists and blockers were seeded at a density of 1 × 10^4^ cells/well in a 96-well flat-bottom plate and cultured for CCK8 and EdU assays according to the manufacturer’s protocols. CCK8 was detected at 0, 24, 48, 72 and 96 h. For the clonogenic assay, 500 cells/well were plated in 6-well plates and cultured in RPMI-1640 medium containing 10% FBS. When colonies were observed after 2 weeks, the plates were washed with PBS and stained with crystal violet for 15 min. All experiments were performed in triplicate.

### Protein extraction and western blotting

The expression of ADRB1, ADRB2, CREB, p-CREB, MMP-7, GAPDH, β-actin, VEGF, MMP-2, JNK, p-JNK, c-Jun, p-c-Jun, c-Fos, p-c-Fos, Caspase-3, Bcl-2, Bcl-XL, Bax, Rb, p-Rb, ERK, pERK and MMP-9 proteins was detected using western blotting as previously described^33^. All antibodies were purchased from Cell Signaling Technology (Danvers, MA, USA).

### RNA interference and lentivirus transfection

Small interfering RNA (siRNA) targeting ADRB2 and non-targeting control siRNA were purchased from Ruibo Biotech. Lentiviral vectors containing shRNA against ADRB2 (shADRB2) and negative control shRNA (shNTC) were prepared by Heyuan Corporation (Shanghai, China). Two GC cell lines (HGC27 NC and MGC803 NC) in 6-well plates were incubated with condensed virus for 12 h. Spectinomycin (Clontech, USA) was used to select lentivirus-transfected GC cells.

### Chronic stress model and drug administration protocols

Female BALB/c nude mice, 4–5 weeks old, were purchased from the Department of Laboratory Animal Centre of Nanjing Medical University. We used female mice only because male mice were homozygous in one cage and were considered to be under stress conditions^29^. All mice were maintained under specific pathogen-free conditions. In this study, the care, use, and treatment of mice were strictly consistent with the care and use guidelines of the Laboratory Manual of the Institute of Basic Medicine. All procedures were performed under sodium pentobarbital anaesthesia, and all efforts were made to reduce pain. Each mouse was constrained and placed in a well-ventilated 50 ml conical tube. Mice were randomly divided into the following groups: control + PBS (*n* = 6), control + propranolol (*n* = 6), control + norepinephrine (*n* = 6), control + epinephrine, stress + PBS (*n* = 6), stress + propranolol (*n* = 6), stress + norepinephrine (*n* = 6) and stress + epinephrine (*n* = 6). To further verify the effect of chronic stress on the proliferation of tumour cells by activating ADRB2 in vivo, we divided nude mice implanted with HGC27 cells into the following groups: (1) stress + PBS (*n* = 6), (2) stress + propranolol (*n* = 6), (3) stress + ICI118,551 (*n* = 6), (4) stress + atenolol (*n* = 6), and (5) stress + terbutaline (*n* = 6). Mice in the stress group were subjected to physical constraint for 8 h per day (restraint stress, from 09:00 to 17:00) 7 days prior to the start of tumour cell injection for a total of 35 days. The tubes were rinsed and sanitized between each restraint cycle. During rest, the mice were provided free access to food and water. Epinephrine (Adr, 2 mg/kg/day), norepinephrine (NA, 3 mg/kg/day), dobutamine (DO, 2 mg/kg/day), terbutaline (TB, 2 mg/kg/day), ICI118,551 (ICI, 5 mg/kg/day) and atenolol (ATE, 5 mg/kg/day) were injected subcutaneously or intraperitoneally. The t_1/2_ of propranolol is 4~5 h in humans but unknown in mice. To prolong the absorption time of propranolol in the body, we formulated slow-release vehicles according to Ben-Eliyahu’s method^44^. Propranolol was dissolved in PBS and added to a mixture of mineral oil (Sigma) and mannide monooleate (a non-specific surface-active emulsifier; Sigma) at a 4:3:1 ratio to create a slowly absorbed emulsion. Propranolol was injected subcutaneously (5 mg/kg in a 0.5 mg/ml concentration) and administered orally (0.1 g/L).

### Measurement of norepinephrine, epinephrine and cortisol levels

Blood samples from nude mice were collected and centrifuged for 15 min at 4000 × *g* and 4 °C. The resultant plasma was maintained at −80 °C until testing. Serum epinephrine, norepinephrine, and cortisol levels were examined using an enzyme immunoassay kit according to the manufacturer’s instructions (Rapidbio Company, Shanghai, China). In brief, the absorbance was measured at 450 nm using a microplate reader. The concentrations of norepinephrine and epinephrine are expressed as picograms per millilitre (pg/ml) of plasma. Each sample was analysed in triplicate.

### In vivo tumourigenesis and metastasis assays

MGC803 cells were implanted by subcutaneous injection of 2 × 10^6^ cells in 100 μl of PBS into the flanks of mice to create tumours. Four weeks later, the mice were sacrificed, and the tumours were harvested. The tumours were cut into two parts. One part was immediately fixed in formalin for TUNEL (Roche, USA) and Ki-67 assays. The other part was prepared for western blotting using a protein extraction kit (KeyGene, Nanjing, China) to analyse VEGF, MMP-2, MMP-7 and MMP-9. Xenograft diameters were measured using a slide calliper every other day until day 28. The xenograft tumour volume was measured twice or three times weekly and was calculated using the following formula: *V* = 1/2 × *ab*^2^ (*a* = the long diameter of the tumour, *b* = the short diameter of the tumour, and *V* = volume). Mouse body weight was monitored throughout the experiment. The diameters of the adrenal glands of the control group and stress group were measured after 4 weeks.

HGC803 cells (1 × 10^6^ cells in 100 μl PBS) were injected into the tail vein of nude mice. After 6 weeks, an IVIS Imaging system (Caliper Life Sciences, Hopkinton, MA) was used to observe the occurrence of distant metastases. The care of experimental animals was in accordance with the Nanjing Medical University Institutional Animal Care and Use Committee.

### Immunohistochemical analysis

Paraffin-embedded, formalin-fixed tissues were immunostained for Ki-67, ADRB2 (Abcam, Cambridge, UK; 1:200 dilution), and PCNA proteins as previously described^33^.

### TUNEL detection assay

A cell apoptosis detection kit (Roche, USA) was used for a TdT-mediated dUTP nick end labelling (TUNEL) assay. Sections prepared from a paraffin-embedded mass were hydrated with an ethanol gradient (100% for 5 min, 100% for 3 min, 95% for 3 min, 85% for 3 min, 70% for 3 min, and 50% for 3 min), fixed with 4% formaldehyde solution, and then incubated with proteinase K for 15 min at room temperature. We used 3% hydrogen peroxide (H_2_O_2_) to block endogenous peroxidases. TUNEL reaction solution containing rTdT was prepared temporarily according to the manufacturer’s protocol. After the slices were washed with PBS, they were counterstained with haematoxylin. Apoptotic cells within the slices were detected with a microscope (Nikon, Japan).

### Samples and patients

GC tissue samples and adjacent normal tissues were collected from 100 patients who underwent radical resection at the First Affiliated Hospital of Nanjing Medical University. No chemotherapy or radiation therapy was administered before surgery. Written informed consent was obtained from all patients or from their relatives. The use of all tissue blocks for this study was approved by the Ethics Committees of Nanjing Medical University. The Ethics Committee permission number is 2015-SRFA-027.

### Statistical analysis

SPSS21.0 was used to analyze the data. The data are presented as the mean ± standard error of the mean (SEM) unless otherwise indicated. If normally distributed, continuous variables between two groups were compared with Student’s *t* test, whereas comparisons between three or more groups were performed with one-way analysis of variance (ANOVA) followed by Tukey’s test. Otherwise, nonparametric tests, such as Tukey’s test, if appropriate, were used to compare differences. Results were considered statistically significant at *p* < 0.05.

## Supplementary information

Supplementary Figure legends

Supplementary Fig.1

Supplementary Fig.2

Supplementary Fig.3

Supplementary Fig.4

Supplementary Fig.5

Supplementary Fig.6
