# Supplementary material for: Chronic stress promotes gastric cancer progression and metastasis: an essential role for ADRB2
Source: Cell Death Dis. 2019 Oct 17;10(11):788. doi: 10.1038/s41419-019-2030-2 (PMC6797812; doi:10.1038/s41419-019-2030-2)
Supplement: Supplementary file 1 — Supplementary Figure legends [file 41419_2019_2030_MOESM1_ESM.docx]

**Supplementary Figure 1** **A-D** CCK8 was used to detect the cell viability of HGC-27 and MGC-803 cells treated with different concentrations of catecholamines. **E,H** CCK8 detection of different concentrations of non-specific ADRB agonists and blockers and treated HGC-27 cell activity. **F,G,I,J** CCK8 was used to detect the activity of HGC-27 cells treated with different concentrations of specific ADRB agonists.

**Supplementary Figure 2** **A** PCNA as a marker of cell proliferation was detected by immunochemical staining. Original magnification, 100×; Scale bar=100μm. **B** Evaluation of DNA synthesis using a EdU test in MGC-803 cells. **C** The percentage of cells positive for PCNA staining is presented **D-G** Pretreatment of HGC27 and MGC803 cells with ADRB1 blocker ATE or ICI or ERK inhibitor U0126 or JNK inhibitor SP600125 prior to adrenaline or terbutaline culture. **H** HGC27 and MGC803 cells were cultured with epinephrine in the absence or presence of ATE, ICI, U0126 or SP600125, and the cell lysate was homogenized to detect p-ERK1/2 and p-JNK expression levels by Western blotting.

**Supplementary Figure 3** Blocking and activating ADRB in different subtypes affected activation of transcription factors NF-κB, AP-1, CREB and STAT3 and regulated the expression of ERK and JNK/MAPK as shown by western blotting in HGC27 and MGC803 cells.

**Supplementary Figure 4 A,B** The use of specific ADRB agonists to pretreat HGC-27 and MGC803 cells obviously attenuated the inhibitory effect of propranolol and ICI 118,551 as shown by a CCK-8 assay. **C,D** Knockdown of ADRB2 in cell lines and CCK8 validation of various types of ADRB agonist on the proliferation of GC. **E** Using the shRNA lines in vivo to confirm the role of ADRB2 in the tumor response to stress exposure. **F** Tumor volumes on the indicated days are presented as growth curves. **G** The mean tumor weight of each group was determined. **H,I,J,K** Pretreatment of HGC27 and MGC803 GC cell lines with propranolol and ICI 118,551 could attenuated the potentiation of epinephrine and norepinephrine by CCK-8 assay and colony forming assay.

**Supplementary Figure 5** **A,B** Effect of ADRB signaling on apoptosis of MGC803 GC cells. **C** The expression of apoptotic key proteins in each treatment group was detected. **D** Western blot analysis showed that CyclinD1/CDK4/CDK6/p-Rb proteins were expressed in each treatment group. **E,F** The percentages of G0-G1 phase in cells treated with specific ADRB2 antagonist ICI118,551 and non-specific ADRB antagonist propranolol were increased

**Supplementary Figure 6** Adrenergic neurotoxic agent 6-hydroxydopamine selectively damages peripheral sympathetic nerves in mice and observes the effect of chronic stress on tumorigenicity in nude mice. **A** From the stress group and the control group are shown.**B** Mean tumor weight. **C** The volume of the tumor during the process of tumor growth at 6 different time points.
