# Supplementary figures and images for: Chronic stress promotes gastric cancer progression and metastasis: an essential role for ADRB2
Source: Cell Death Dis. 2019 Oct 17;10(11):788. doi: 10.1038/s41419-019-2030-2 (PMC6797812; doi:10.1038/s41419-019-2030-2)

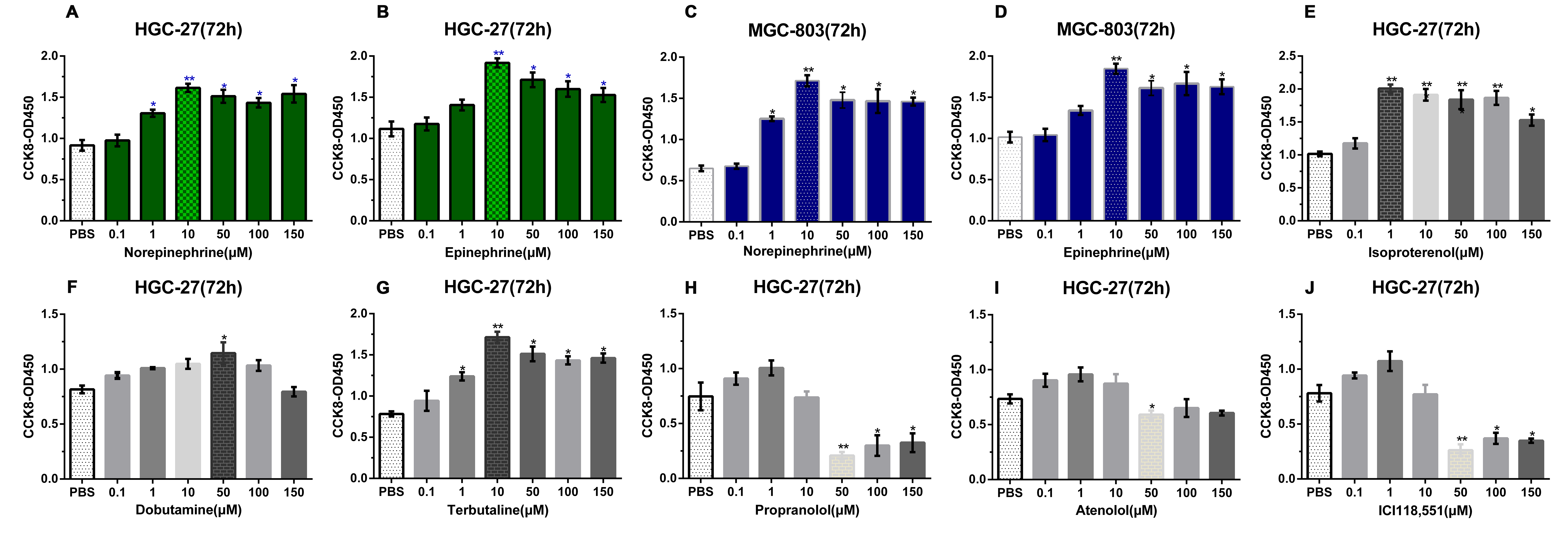

Supplement: Supplementary file 2 — Supplementary Fig.1 [file 41419_2019_2030_MOESM2_ESM.tif]

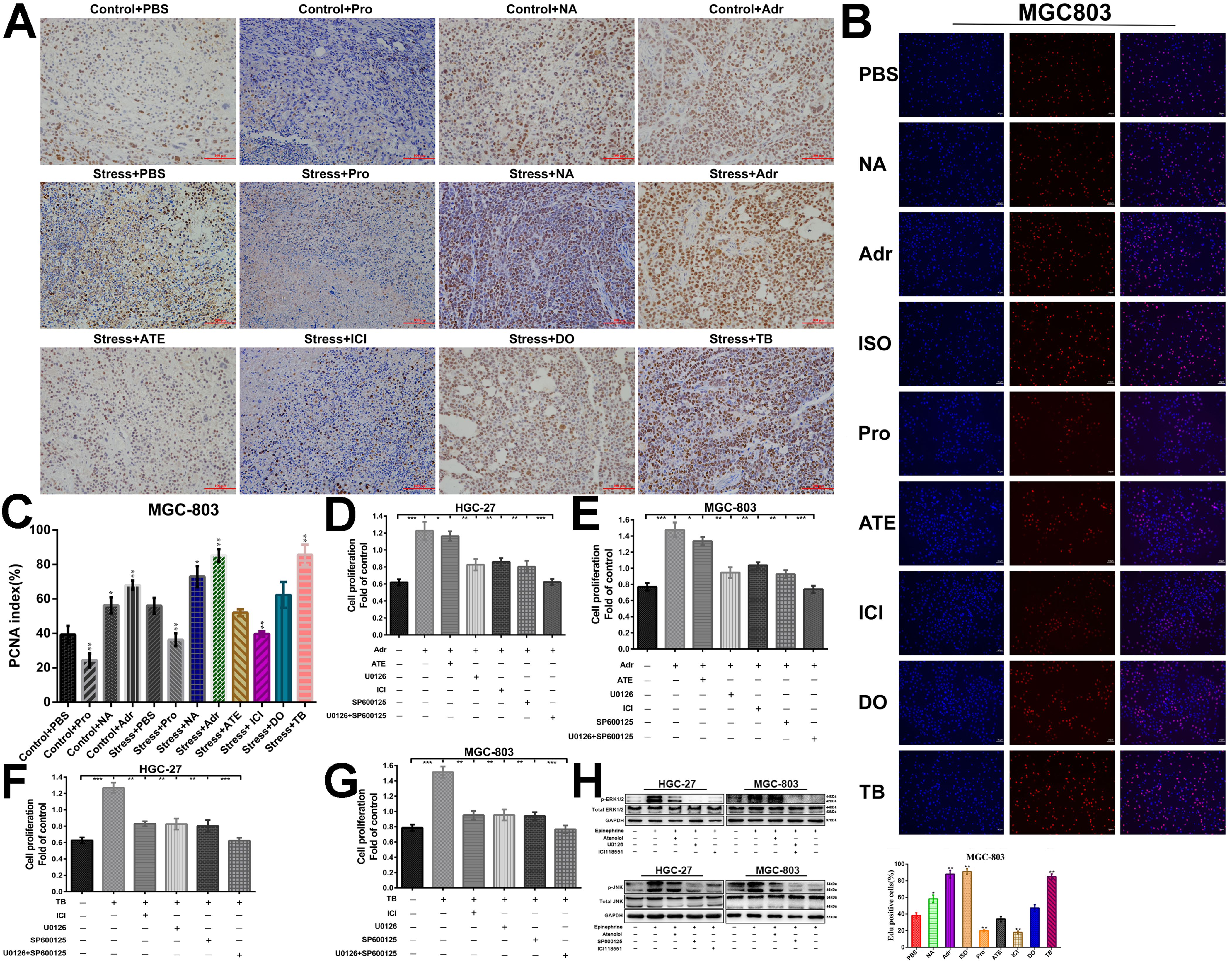

Supplement: Supplementary file 3 — Supplementary Fig.2 [file 41419_2019_2030_MOESM3_ESM.tif]

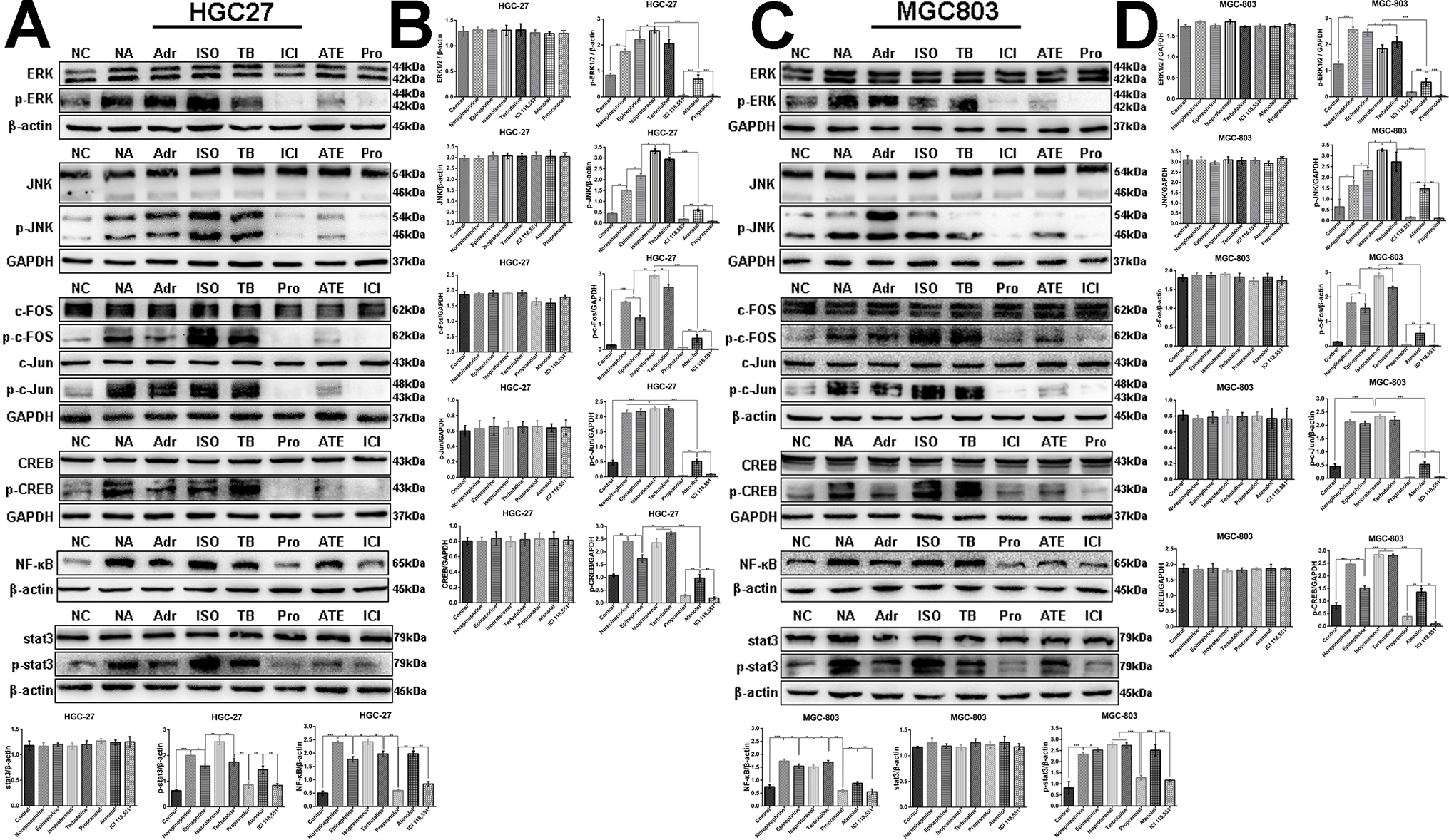

Supplement: Supplementary file 4 — Supplementary Fig.3 [file 41419_2019_2030_MOESM4_ESM.tif]

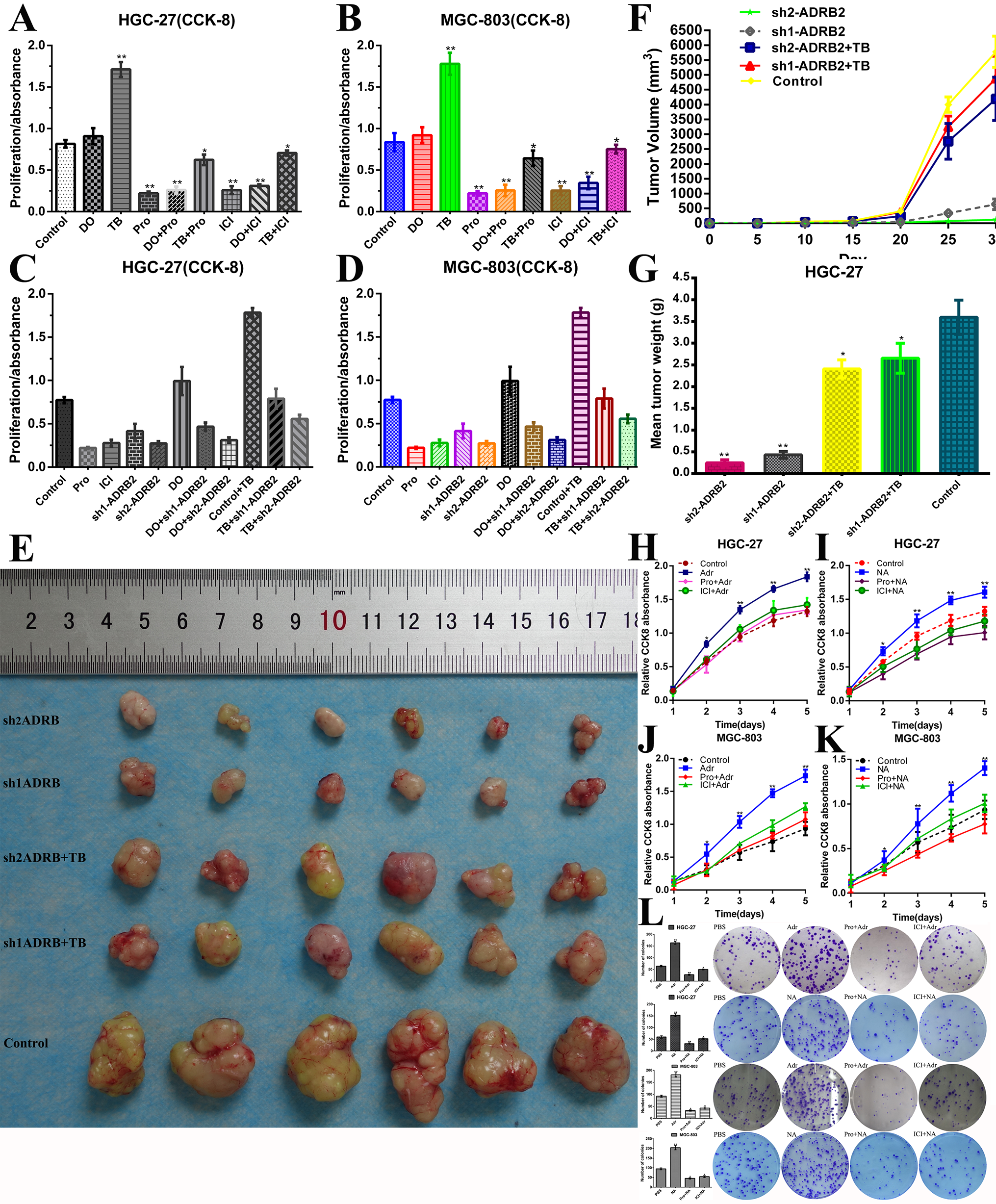

Supplement: Supplementary file 5 — Supplementary Fig.4 [file 41419_2019_2030_MOESM5_ESM.tif]

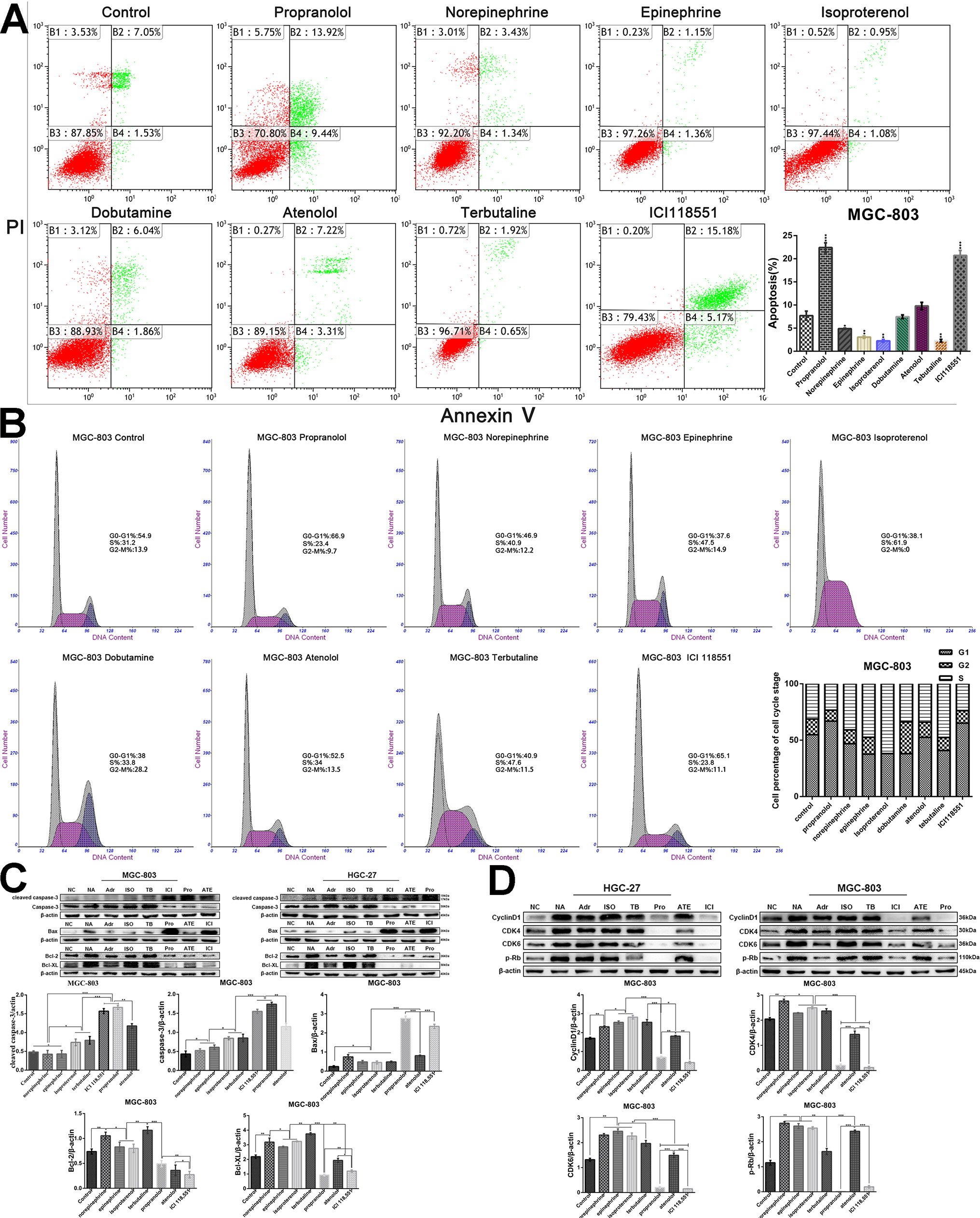

Supplement: Supplementary file 6 — Supplementary Fig.5 [file 41419_2019_2030_MOESM6_ESM.tif]

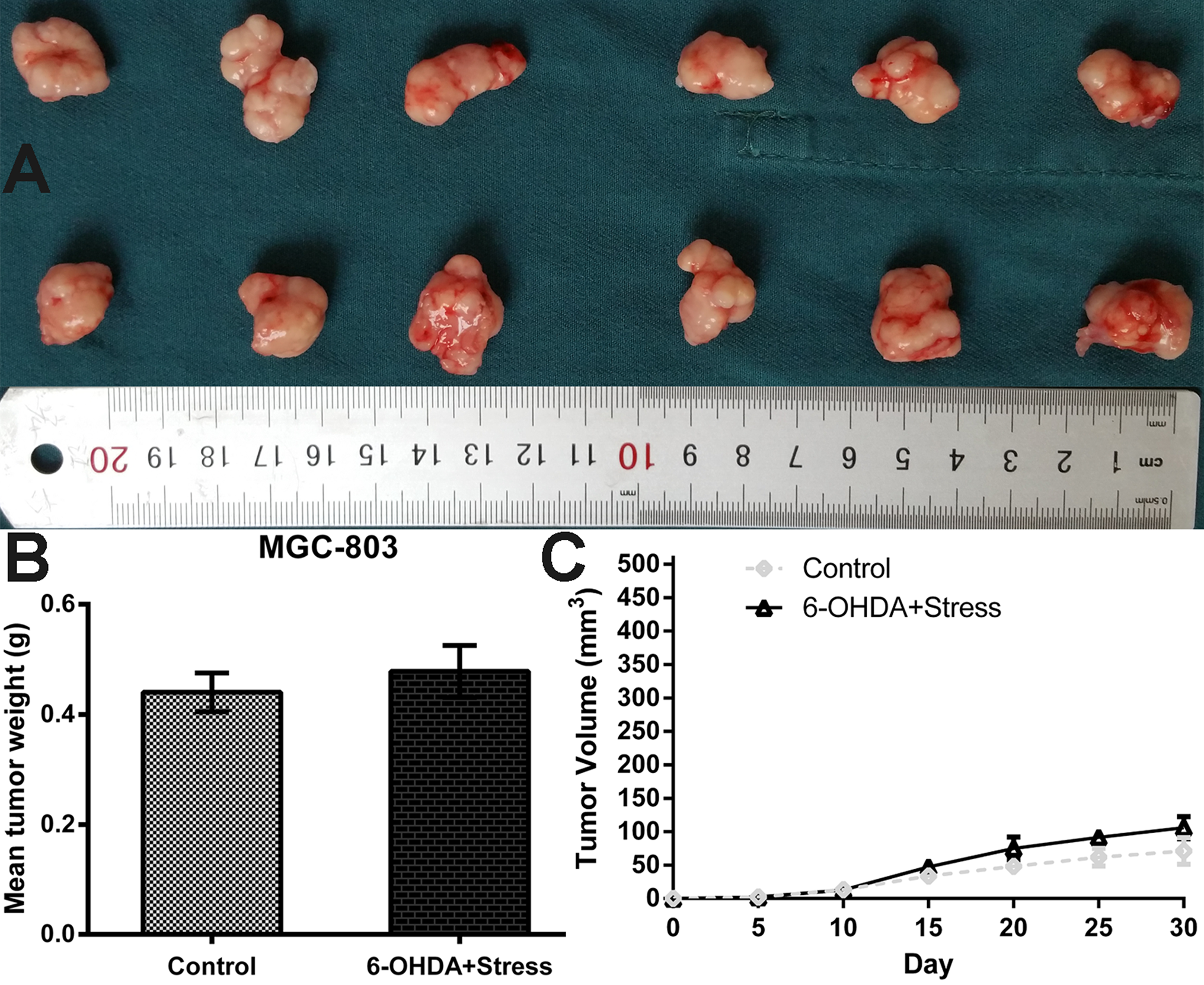

Supplement: Supplementary file 7 — Supplementary Fig.6 [file 41419_2019_2030_MOESM7_ESM.tif]
